# Determinants of maternal mortality among obstetric patients admitted to intensive care unit of Wolaita Sodo comprehensive specialized hospital, Southern Ethiopia: unmatched case-control study

**DOI:** 10.1186/s12884-025-07979-1

**Published:** 2025-08-23

**Authors:** Tigist Nega Alemu, Wondimagegn Genaneh Shiferaw, Wakgari Binu Daga, Yordanos Sisay, Behailu Balcha

**Affiliations:** 1 Department of Emergency and Critical Care Nursing, Wolaita sodo University, Sodo City, Ethiopia; 2 School of Public Health, Wolaita Sodo University, Sodo City, Ethiopia

**Keywords:** Maternal mortality, Intensive care unit, Unmatched case control, Wolaita Sodo

## Abstract

**Background:**

Maternal mortality among obstetric patients admitted to intensive care units (ICUs) remains disproportionately high in low-resource settings. Limited evidence exists regarding its determinants in such contexts. This study aimed to identify factors associated with maternal mortality among obstetric patients admitted to the ICU of Wolaita Sodo University Comprehensive Specialized Hospital, Southern Ethiopia.

**Methods:**

An unmatched case–control study was conducted from November 23 to December 23, 2023, at Wolaita Sodo University Comprehensive Specialized Hospital, Southern Ethiopia. A total of 378 obstetric ICU admissions over a 10-year period were reviewed, including 126 maternal deaths (cases) and 252 survivors (controls), selected using simple random sampling. Data were extracted using a structured, pretested checklist. Bivariate analysis was used to identify candidate variables (*p* < 0.25) for inclusion in multivariable analysis where in statistical significance was established at *p* < 0.05. Strength of association was expressed by Adjusted odds ratios (AOR) with 95% confidence.

**Results:**

Significant predictors of maternal mortality included ICU stay of fewer than five days [AOR = 2.61; 95% CI: 1.41–4.82], use of invasive mechanical ventilation [AOR = 3.82; 95% CI: 1.90–7.30], presence of shock [AOR = 6.80; 95% CI: 3.24–14.14], and multi-organ failure [AOR = 2.69; 95% CI: 1.03–6.98]. Mild [AOR = 0.19; 95% CI: 0.09–0.43] and moderate [AOR = 0.08; 95% CI: 0.04–0.18] reductions in the level of consciousness on admission were associated with lower odds of mortality compared to severe reduction.

**Conclusion and recommendations:**

Shorter ICU stay, invasive mechanical ventilation, development of shock, and multi-organ failure were independently associated with increased maternal mortality. Improved ICU triage protocols, early identification of complications, and appropriate critical care interventions are essential to improve maternal outcomes.

## Introduction

The World Health Organization defines maternal mortality as any form of death related to pregnancy or pregnancy management, excluding an accidental or incidental cause that occurs during pregnancy or within 42 days of the end of the pregnancy [1, 2]. Maternal mortality, a prominent marker of global disparity within the healthcare system, is characterized by an alarmingly high incidence [3]. The presented statistics reveal a dearth of accessible and superior quality health services, potentially indicative of insufficient policy implementation aimed at safeguarding women's health [4, 5]. The majority of maternal mortalities could be alleviated if high-quality services are delivered for women in entire healthcare systems [1, 6]

Obstetric patients are frequently young and healthy and also often out of obstetric incidents [7]. Nevertheless, a few proportions of these women will be granted admission to the intensive care unit (ICU), and for some of these women, admission to the intensive care unit (ICU) can be a matter of life and death [8]. The rate of admission to intensive care may be considered an objective indicator of severe maternal morbidity and the mortality from such admissions is very high [9, 10]. The commonest obstetric cases that lead to ICU admission include massive hemorrhage, hypertensive disorders, sepsis, thromboembolism, acute organ dysfunction, and anesthesia-related [11–13]. Whereas hypertensive disorders of pregnancy is leading cause of maternal mortality [14–16]. Obstetric hemorrhage, decreased consciousness, lower educational attainment, complications from severe hypertension, delayed hospital admission, multiple organ failure, sepsis, and lack of access to prenatal care are other significant factors that contribute to maternal mortality in intensive care units [17, 18]

ICU maternal mortality rates are much higher in under-resourced nations than in developed ones, even though the demographics of obstetric patients admitted to these units are similar [13, 19]. The ICU mortality rate in low-income countries ranges from 2 percent to 43 percent, while in developed countries it ranges from 0 percent to 4 percent [20]. ICU maternal mortality rate in Africa particularly had reached a concerning 30.69% nearly ten times higher than developed world [21]. Ethiopia is one of the peak maternal death-reporting countries in sub-Saharan Africa, with an annual mortality rate of 401 per 100 000 live births [15]. Ethiopia has shown a significant reduction (71%) in maternal mortality but experienced significant maternal and child mortality from preventable causes [22]. Some of challenges for maternal health in Ethiopia and the whole Eastern Africa are poor quality of healthcare, poor infrastructure and policies with deprived focus on maternal health [6].

The World Health Organization (WHO) with other stakeholders has released an agreement statement and strategy paper on ending preventable maternal mortality aiming to reach global maternal mortality rate less than 70 maternal deaths per 100 000 live births by 2030 [23]. The transition from MDG to the SDG in 2015 led to amplified concentration on the continuum of care for women, thus including critical care [24]. In surveys which were done in various countries showed that the use of high-quality ICU service is remarkably correlated with decrement in MMR [25]. In order to prevent future deaths and to address all causes of maternal mortality, it be mandatory to comprehend the immediate and underlying causes of maternal deaths and to develop evidence-based, context-specific program interventions [26]. Systematically evaluating every maternal death is one of the strategies to accomplish this goal. Not only, has it allowed the identification of the number of maternal deaths but also the linked possible causes and contributors [27, 28].

The country had established the Maternal Death Surveillance and Response (MDSR) system in 2013 to make actual data that inform decision-making by providing insights into the roots and contributing factors of maternal deaths [15]. But evidences stated in a death review thus represents a partial and contestable version of occasions [29]. Despite this discrepancy little research has been conducted in the country, particularly the case of ICU, Under possibly assessed journals and web sited there is no study that assessed maternal mortality in ICU of the study area even in the region at large. Thus, this study aimed to close identified gaps by determining the factors that contribute to maternal mortality among obstetric patients admitted to intensive care unit of Wolaita Sodo Comprehensive Specialized Hospital (WSUCSH) by incorporating the most significant variables. It will also close existing knowledge gaps, highlight care breaches in the study area, and serve as a framework for future research [27, 28].

## Methods and materials

### Study area and period

The study was carried out at WSUCSH from November 23 to December 23, 2023 using 10 years period data. The hospital is located in South Ethiopia's Wolaita zone, in the town of Wolaita Sodo. The distance between Wolaita Sodo City and Addis Ababa, Ethiopia's capital, is 330 kilometers southwest. One of the 7 departments is the Obstetrics and Gynecology department, which has 6 wards. Amongst the services offered by the obstetrics and Gynecology departments are delivery, postnatal care, antenatal care, family planning, EPI, comprehensive abortion care, gynecological surgeries and procedures, PACU, Gynecology OPD, cervical cancer screening, and NICU service [30]. The hospital has a single 10 bed adult ICU which serves for all critically ill patients of all departments including sever Obstetrics cases from different Woredas’. 

### Study design

An institutional-based unmatched case-control study design was carried out.

### Source and study population

#### Source of population

All obstetrics patients admitted to ICU of WSUCSH from November 2014 to November 2023.

#### Study population

Case; All obstetric patients hospitalized to the ICU of WSUCSH in the 10 years period, whose pregnancy period is more than 28 weeks and within 42 days following delivery due to obstetric or co-morbid conditions and who were died after admitted to ICU.

Control; All obstetric patients hospitalized to the ICU of WSUCSH in the 10 years period, whose pregnancy period is more than 28 weeks and within 42 days following delivery due to obstetric or co-morbid conditions and who discharged alive.

### Inclusion and exclusion criteria

#### Inclusion criteria

All obstetric patients, whether alive or dead, were admitted to the intensive care units of Wolaita Sodo University Comprehensive Specialized Hospital during a ten-year period and whose pregnancies lasted longer than 28 weeks and within 42 days after delivery because of obstetric or co-morbid conditions.

#### Exclusion criteria

Pregnant or newborn mothers whose medical records were incomplete (more than 10 percent of missing data) which means that important information such as patient outcome status, socio-demographic information, and tried treatment interventions were missed from chart. Pregnant or postpartum mothers who were admitted for any accident (road traffic accident, accidental injury, poisoning, etc.) other than an accident that is the subject of treatment (uterine or cervical tear, drug-related complication). Obstetric patients who were referred to other hospitals for further investigations or treatments that make it difficult to identify the final outcome of the treatment.

### Sample size

The sample size was calculated by Epi info software version 7 using an unmatched case-control Fleiss formula from the case-control ICU study of Addis Ababa Public hospitals by checking different determinant variables. Puerperal sepsis is found to give the highest sample size by considering confidence level of 95%, power 80% and case to control ratio of 1:2 and results maximum sample size with a correction factor of 468 [17]. The final sample size results 468, which are 156 cases and 312 controls.

 Power = 80%, 𝒁𝜷 = 0.84 for 20% beta error.

 𝒑1 = proportion of cases with exposure = 22.7%

 𝒑2 = proportion of controls with exposure = 12%

r = case to control ratio (1case to 2 controls) = 1:2

𝒏1_Fleiss-cc_ = required sample size for cases using Fleiss's formula with continuity correction.

𝒏1= sample for cases, 𝒏2 = sample for controls. 𝒏2=2 𝒏1or 1:2 ratio

 The sample size formula withthe correction factor by Fleiss is:

$$n_1cc=\frac{n_1}4\left[1+\sqrt{1+\frac{2\left(r+1\right)}{n_1r\left|p_1-p_2\right|}}\right]^2=156$$

$$n2=2n1\;then\;\;n\_2=156\times2=312\;$$

The final a total sample size of 156 + 312= 468; see Table 1 for detailed information)

**Table 1:** Sample size Estimation based on key clinical determinants of maternal mortality among obstetric ICU admissions at WSUCSH, 2024

Variables	Proportion of cases exposed		Proportion of control exposed	Cases from sample size with cc	Controls from sample size with cc	Total sample size With cc	Reference
Sever preeclampsia		34.7%	14%	55	110	165	[17]
Absence of ANC follow up		14.7%	6%	153	305	458	[17]
Puerperal sepsis		22.7%	12%	156	312	468	[17]
Co-morbidity		38.7%	15.3%	46	92	138	[17]
GCS < 9		24%	8%	67	133	200	[17]
GCS 9–12		38.7%	23.3%	103	206	338	[17]

### Sampling technique

A total of 489 patient charts were retrieved from the ICU registration book, representing obstetric cases admitted over a decade. The charts of both alive and dead patients were documented separately, and a simple random sampling method (using a lottery method) was employed to select study subjects, totaling 468 cases. For each selected case, 2 controls were also included. Ultimately, 378 patients’ charts (81%) met the established inclusion criteria.

### Variables of the study

- Dependent variables
  - ➢ Obstetrics patients outcome status (Alive vs. died)
- Independent variables
  - ➢ Socio-demographic factors (age, residence and marital status)
  - ➢ Patient-related Factors (Gravidity, Parity, Maternal co-existing medical illness, Vital sign at admission, GCS or level of consciousness, Presentation at admission and Obstetrics reasons for admission)
  - ➢ Treatment-related factors (ANC follow up, Treatment delivered in ICU, length of ICU stay and complications encountered in ICU)

### Operational definition

Maternal mortality: is the death of a woman while pregnant or within 42 days of termination of pregnancy, irrespective of the duration and the site of the pregnancy, from any cause related to or aggravated by the pregnancy or its management, but not from accidental or incidental cause which was defined using WHO ICD-10 criteria [31].

 Case: a pregnant woman of gestational age >28 weeks or delivered mother within 42 days post-delivery who has died due to obstetric issues or from developed complications after being admitted to ICU [17].

Control: a pregnant woman of >28 weeks gestational age or delivered mother within 42 days of post-delivery who got discharged from ICU after improving from obstetric disease conditions or its developing complications [17].

Length of stay in ICU: in this study, length of stay is the time expressed in days, when obstetric patients stayed in ICU from admission to the occurrence of death or discharged by a working physician. 

Complications developed in ICU: in this study complications are a disease conditions comprises of Shock, Multi organ failure, Healthcare associated infections and disseminated intravascular coagulopathy that the women didn’t diagnose during ICU admission and believed to have been acquired while under ICU treatment. 

Multi-Organ Failures (MOFs):in this study multi-organ failure is the presence of more than one diagnosed organ (Lung, Heart, Brain etc.) failure after admission to ICU.

Decreased level of consciousness: is reducedlevel responsiveness of patients which was measured up on admission by using Glasgow coma scale with lowest value of 3 and highest value of 15, interpreted as a severely decreased level of consciousness (GCS of 3-8), moderately decreased level of consciousness (GCS of 9-12) and mildly decreased level of consciousness (GCS of ≥ 13)

### Data collection tool and procedure

The data was collected from patients’ paper charts or cards using a developed data extraction checklist. The checklist was developed after reviewing prior published studies [1, 2, 11, 14, 17, 20]. The checklist has six parts with a total of sixty-two questions. The first part contains socio-demographic- characteristics, the second part contains patient-related factors, the third part contains clinical characteristics, the fourth contains treatment-related, the fifth part contains health related parameters, and the six parts contain complication-related factors. The data were collected by two trained staff members at WSUCSH from selected patients'reasonably complete (≥ 90 percent of variables) paper charts obtained from liaison office. The overall completeness of the data was 96.4%.

### Data quality assurance

The questionnaire was prepared in English. Training and orientation about the objectives and relevance of the study on each item included in the study tools and the whole process of data collection was provided by data collectors and supervisors. The protest was occurring in WSUCSH on 24 (8 cases and 16 controls) client charts, i.e. 5 % of the sample size before the actual data collection and were excluded from the final analysis. During data collection, regular supervision and follow-up was undertaken. The supervisor checked each collected questionnaire daily. Further cross-checked by the principal investigator and is entered into statistical software in care full manner to minimize errors.

### Data processing and analysis

Data was checked manually for completeness by the supervisor and then coded and entered into Epi data 4.6 by the investigator carefully. Simultaneously, data cleaning and analysis was conducted using SPSS version 25 by the investigator. The normality test of Kolmogorov-Smirnov (<0.05) was done to check data distribution for continuous variables, then descriptive data was expressed by frequency, percentages and median (with inter-quartile quartile range). The chi-square test is used to compare the observed result with the expected result. A binary logistic regression model was applied to assess the association between the variables. The associated factors are determined by bi-variate and multivariable analysis and those variables that had *P*< 0.25 on bi-variate analysis were directly forwarded to both analysis and control confounders by multivariable analysis. Hosmer and Lemeshow's goodness of fit test for the model fitness (P-value: 0.69) and variables having *p*<0.05 on multivariable analysis were considered statistically significant. The strength of the association between the dependent and independent variables is expressed using an odds'ratio with a confidence interval (CI) of 95%. Finally, the results were presented using texts, tables, and graphs and interpreted into valuable information.

### Ethical consideration

Ethical clearance was obtained from WSUCSH; Ethical review committee with project number: CHSM/ERC/01/16, after being informed by written letter to conduct the study as well as to check patients ‘medical records, then data were collected in a way that makes it impossible, at least very hard, to identify, using code numbers and by keeping questionnaires in a safe place. As a result, the confidentiality of the data is kept safe.

## Results

### Socio-demographic characteristics of the participants: Wolaita Sodo University Comprehensive Specialized Hospital, Wolaita Sodo Ethiopia, 2024

Approximately 378 participants (81% of the calculated sample size) who fulfilled the inclusion criteria were studied; unobtainable charts and charts with missing data (more than 90% of variables) were excluded during data collection time. The age of obstetric mothers was found between 18 and 45 years for cases, while between 18 and 48 for control groups. The median age among the cases was 29 years with an inter-quartile range of 8.25 years. Consistently, 29 years was the median age for controls with an IQR of 5.75 years. Most of the cases, 103 (81.7%) and control 218 (86.5%) age were situated between18- 34 years age group.

The majority of 88 (69.9%) of ICU-admitted cases came from rural areas. In contrast, more than half, 144 (57.2%) of the controls were sourced from urban areas. Around two-thirds of people in both groups were married, while just 3 percent of obstetric cases and controls were widowed; (see Table 2 for more information).

**Table 2:** Sociodemographic characteristics of obstetric patients admitted to ICU, stratified by survival outcome, at WSUCSH, 2024

Variables	Categories	Outcomes Cases *n* (%)	Controls *n* (%)	X^2^-Square	COR (95%CI)	*P*-value
Age	18–34	103(81.7)	218(86.5)	1.481	0.698	0.223
	35–48	23(18.3)	34(13.5)			
Residency	Urban	38(30.1)	144(57.2)	7.943	0.523	0.005
	Rural	88(69.9)	138(54.8)			
Marital status	Married	95(75.4)	187(74.2)	0.002	0.986	0.966
	Divorced	10(7.9)	23(9.1)	0.124	0.844	0.725
	Widowed	4(3.2)	9(3.5)	0.048	0.863	0.826
	Single	17(13.5)	33(13.2)			

### Intensive Care Unit admission related parameters of the participants at WSUCSH

Most of the admitted obstetric cases were multigravida, 92(73%), and likewise, 179 (71.1%) were controls. About 57(45.3%) cases and 103(40.9%) controls did not have any ANC follow-up either in the referred institution or in WSUCSH. Overall, a minimum of one day was the lowest length of stay in the ICU, while 63 days was the longest ICU duration. Three days for obstetric cases and 4 days for control groups were the median lengths of ICU stays within their respective inter-quartile range of 3 and 2 days. most study participants i.e. (71.4%) cases and nearly half of controls were stayed for less than 5 days. Greater fractions (65.9%) of cases were postpartum presentations, and merely 5% were post-abortive. Even though half of the controls were postpartum and equally only 5% were post-abortive as compared to cases;(see Table 3 for detailed information)

**Table 3:** Admission-related obstetric and clinical parameters among ICU-admitted obstetric patients by outcome status, WSUCSH, 2024

Variables	Categories	Outcomes Cases *n* (%)	Controls *n* (%)	X^2^-Square	COR (95%CI)	*P*-value
Gravidity	Primigravida	34(27)	73(28.9)	0.163	0.906	0.686
	Multigravida	92(73)	179(71.1)			
Parity	Primipara	50(39.7)	90(35.7)	0.567	0.452	0.451
	Multipara	76(60.3)	162(64.3)			
Prior ANC follow ups	Yes	69(54.7)	149(59.1)	0.656	0.837	0.418
	No	57(45.3)	103(40.9)			
Length of stay	< 5 days	90(71.4)	133(52.7)	12.07	2.244	0.001
	≥ 5 days	36(28.6)	119(47.3)			
Presentation at admission	Pregnant	37(29.4)	112(44.5)	0.601	0.661	0.438
	Post-partum	83(65.9)	128(50.8)	0.250	1.297	0.617
	Post abortion	6(4.7)	12(4.7)			

### ICU admission reasons of obstetrics patients admitted to ICU of WSUCSH

**Fig. 1:**
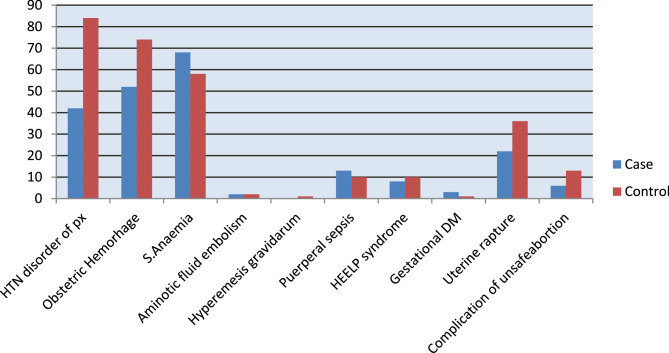
Distribution of primary obstetric conditions leading to ICU admission among obstetric patients at Wolaita Sodo University Comprehensive Specialized Hospital, 2024.Where HTN-Hypertension, S- Sever, HEELP- Hemolysis, Elevated Liver enzymes and Low Platelets, DM- Diabetes Mellitus

Severe anemia 68 (54%), obstetric Hemorrhage 52 (41.2%) and Hypertensive disorder of pregnancy 42 (33.3%) i.e. Severe Preeclampsia (22%) and Eclampsia (78%) were the predominant obstetric reasons for admission to the ICU for cases. Likewise, Severe anemia 129(51.2%), Hypertensive disorder of pregnancy 82 (32.5%) and Obstetric Hemorrhage 72 (28.6%) were predominant obstetric reasons for control groups; (see Fig. 1 for additional information).

**Fig. 2:**
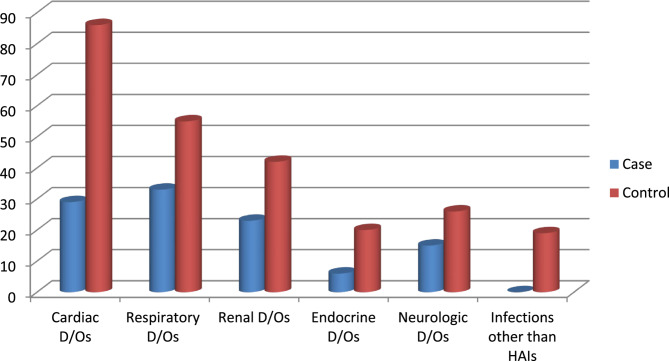
Distribution of non-obstetric medical conditions contributing to ICU admission among obstetric patients at Wolaita Sodo University Comprehensive Specialized Hospital, 2024. Where D/Os- Disorders and HAIs- Healthcare-Associated Infections

The commonest non-obstetric reasons amongst case were respiratory disorders 33(26.1%), Cardiac disorders 29(23.0%) and Renal disorders 23(18.2%), whereas cardiac disorders were the predominant non-obstetric reason of admission 85(34.1%) followed by Respiratory disorders 55(21.8%) and Renal disorders 42(16.7%) amongst controls; (see Fig. 2 for additional information)

### Management modalities of maternal mortality among admitted obstetric patients

Most of the admitted cases, 119(94.4%) and controls 248(98.4%) had taken antibiotics for either prophylaxis or therapeutic purposes; Around 93(73.8%) cases and 159(63%) controls had episodes of taking any of the blood products as a transfusion. Cardio-Pulmonary Resuscitation (CPR) was done to preserve the lives of 18(14.2%) cases and for 28(11.1%) mothers. Dialysis procedures were tried in 13(10.3%) cases and 8(3.1%) controls of ICU admitted obstetric mothers and Mechanical Ventilation support were applied for the majority of cases 101 (80%) and 109(43.2%)of controls; (refer Table 4 for more information).

**Table 4:** Critical care interventions and therapeutic modalities administered to obstetric ICU patients by mortality outcome, WSUCSH, 2024

Variables	Categories	Outcomes Cases*n*(%)	Controls*n*(%)	X^2^-Square	COR (95%CI)	*P*-value
Antibiotics	Yes	119(94.4)	248(98.4)	4.682	0.274	0.030
	No	7(3.7)	4(1.6)			
Blood products transfusion	Yes	93(73.8)	159(63.1)	4.339	1.649	0.037
	No	33(26.2)	93(36.9)			
Anticoagulants	Yes	25(19.8)	54(21.4)	0.128	0.908	0.720
	No	101(80.2)	198(78.6)			
Catecholamine	Yes	17(13.5)	25(9.9)	1.085	1.416	0.298
	No	109(86.5)	227(90.1)			
Inotropes	Yes	1(0.8)	2(0.8)	0.001	1.000	1.000
	No	125(99.2)	250(99.2)			
CPR	Yes	18(14.3)	28(11.1)	0.792	1.333	0.373
	No	108(85.7)	224(88.9)			
Dialysis	Yes	13(10.3)	8(3.2)	8.168	3.509	0.040
	No	113(89.7)	244(96.8)			
Oxygen via Nasal Catheter	Yes	89(70.6)	191(75.8)	1.164	3.509	0.181
	No	37(29.4)	61(24.2)			
Mechanical Ventilation	Yes	101(80.2)	109(43.3)	46.334	5.300	0.001
	No	25(19.8)	143(56.7)			
Manitol	Yes	4(3.2)	6(2.4)	0.205	0.768	0.650
	No	122(96.8)	246(97.6)			
Diuretics	Yes	31(24.6)	54(21.4)	0.486	1.344	0.486
	No	95(75.4)	198(78.6)			
Magnesium Sulphate	Yes	44(34.9)	70(27.8)	0.000	1.000	1.000
	No	82(65.1)	182(72.2)			
Hysterectomy	Yes	7(5.6)	16(6.3)	0.093	0.868	0.761
	No	119(94.4)	236(93.7)			
C/S	Yes	4(3.2)	7(2.8)	0.047	1.148	0.829
	No	122(96.8)	245(97.2)			

### Health status indicator parameters of admitted obstetric patients at WSUCSH

On admission participants ICU base line vital signs were recorded, and about one-third of admitted cases were tachycardic as well as hypertensive. Simultaneously, their admission level of consciousness was assessed using Glasgow Coma Scale and minimum GCS were 3 while 15 were the maximum, resulting range of 12. Ten were the median with ICR of Seven. Thirty-eight percent of the obstetric patients were unconscious during (3-8 GCS) admission; (see Table 5 for more information).

**Table 5:** Baseline physiological status and level of consciousness at ICU admission among obstetric patients, stratified by outcome, WSUCSH, 2024

Variables	Categories	Outcomes Controls *n* (%)	Cases *n* (%)	X2-Square	COR (95%CI)	*P*-value
Base line PR	Bradycardia	34(13.5)	22 (17.4)	0.168	1.149	0.682
	Normal	147(58.3)	64(50.8)	1.080	0.773	0.299
	Tachycardia	71(46.2)	40(42.8)			
Base line BP	Hypotension	47(80.1)	23(19.8)	0.029	0.948	0.864
	Normal	112(74.6)	55(26.4)	0.042	0.951	0.837
	Hypertension	93(66)	48(34.0)			
Baseline saturation	Normal	129(68.3)	60(31.7)	0.429	1.154	0.585
	Hypoxia	123(65.1)	66(34.9)			
Baseline GCS	3–8	54(37.5)	90(62.5)			
	9–12	98(89.1)	12(10.9)	55.34	0.073	0.001
	≥ 13	100(80.6)	24(19.4)	46.19	0.144	0.001

Where: *PR *Pule Rate, *BP B*lood Pressure, *GCS *Glasgow Comma Scale

### Complications encountered among ICU admitted obstetric patients at WSUCSH

Shock was the most common ICU complication amongst cases 82 (65%). Of these, about (68) 83% were Hypovolemic Shock, followed by Septic Shock 18 (23%). Even as, amongst control groups, almost one-third had developed shock, from these 75 (91.5%) were Hypovolemic which is followed by septic shock of 10 (12%).

**Fig. 3:**
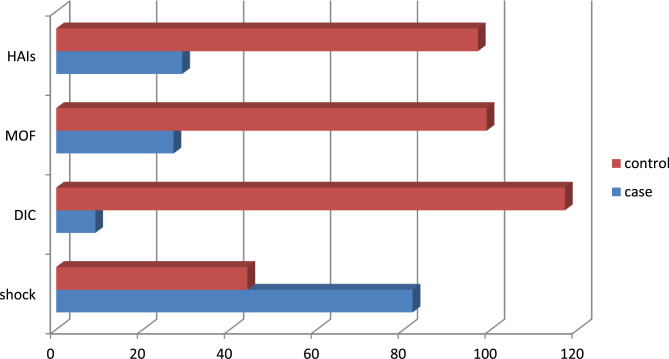
Types and frequency of complications developed during ICU stay among obstetric patients at Wolaita Sodo University Comprehensive Specialized Hospital, 2024. Where HAI–Hospital acquired infection MOF- Multi organ failure DIC-Disseminated Intravascular Coagulopathy

Shock was the most common ICU complication amongst cases 82 (65%). Of these, about (68) 83% were Hypovolemic Shock, followed by Septic Shock 18 (23%). Even as, amongst control groups, almost one-third had developed shock, from these 75 (91.5%) were Hypovolemic which is followed by septic shock of 10 (12%) (Fig. 3).

### Determinant factors maternal mortality among ICU admitted obstetric patients of WSUCSH, Southern Ethiopia, 2024

After bi-variate analysis has carried out, the following variables (Fourteen) are considered to be statistically significant with maternal mortality. These are age, residency, length of stay in ICU, obstetric Hemorrhage, HEELP syndrome, Gestational Diabetes Mellitus, Cardiac disorders, took Antibiotics, and Transfusion of blood products, carried out Dialysis, had been on invasive Mechanical Ventilation, altered level of consciousness (GCS), developed Shock and developed Multi-Organ Failure complications.

Multivariable analysis has verified that 5 variables are determinants of maternal mortality among ICU-admitted obstetric patients of WSUCSH. These factors are Length of stay (1-4 days), Being on invasive Mechanical Ventilation, decreased admission level of consciousness (GCS) of moderate (9-12) and mild (13-15), patients with Multi-Organ Failure (MOF) and Shock.

Odds of maternal mortality was nearly 3 time [AOR=2.61, 95% CI: 1.41, 4.82] higher for obstetric patients who stayed in ICU for <5 days as compared to those stayed in the ICU for more than 5 days.

Odds of maternal mortality was nearly 4 times [AOR=3.82, 95% CI: 1.9, 7.3] higher among those on invasive Mechanical Ventilation as compared to with those participants who were not supported by the machine.

Odds of maternal mortality was about 80 percent [AOR=0.19, 95% CI: 0.09, 0.43] less for mildly (13-15) decreased level of consciousness (GCS) on admission and 92 Percent [AOR=0.08, 95% CI: 0.04, 0.18] less for moderately (9-12) decreased level of consciousness on admission as compared to severely decreased level of consciousness.

Odds of maternal mortality was nearly 7 time [AOR=6.8, 95% CI: 3.24, 14.14] higher for obstetric patients whom developed shock in ICU in comparison with mothers who were not diagnosed with shock as a complication.

Odds of maternal mortality was nearly 3 times [AOR=2.69, 95% CI: 1.03, 6.98] higher for obstetric patients whom developed Multi-Organ Failure (MOF) as compared to those mothers who weren’t developed Multi-Organ Failure (MOF); (see Table 6 for more information)

**Table 6:** Multivariable logistic regression analysis of independent predictors of maternal mortality among obstetric ICU admissions at WSUCSH, 2024

Variables	Category	Case	Control	COR (95% CI)	*P*-value	AOR(95%CI)	*P*-value
Age	18–34 yrs. ≥35 yrs.	218 34	103 23	0.69[0.39–1.24] 1	0.224	0.92[0.42–2.01] 1	0.841
Residency	Urban Rular	144 138	38 88	0.52[0.33–0.82] 1	0.005	0.68[0.37–1.25] 1	0.224
Length of ICU stay	< 5 days ≥ 5 days	90 36	133 119	2.24[1.41–3.54] 1	0.001	2.61[1.41–4.82] 1	0.002
Obstetric hemorrhage	Yes No	52 74	72 180	1.7[1.12–2.74] 1	0.014	1.54[0.74–3.18] 1	0.243
HEELP syndrome	Yes No	10 116	8 244	2.62[1.02–6.83] 1	0.047	2.85[0.83–9.76]	0.096
Gestational DM	Yes No	3 123	1 251	6.12[0.63–59.46] 1	0.118	9.46[0.37–23.9] 1	0.174
Cardiac disorders	Yes No	29 97	86 166	0.57[0.35–0.94] 1	0.028	0.97[0.49–1.90] 1	0.934
Taking antibiotics	Yes No	119 7	248 4	0.27[0.07–0.95] 1	0.042	0.34[0.07–1.59] 1	0.178
Blood transfusion	Yes No	93 33	159 93	1.64[1.02–2.64]	0.038	0.50[0.22–1.12] 1	0.099
Dialysis	Yes No	13 113	8 244	3.50 = 1.41–8.70] 1	0.007	1.11[0.35–3.51] 1	0.851
IMV	Yes No	101 25	109 143	5.30[3.20–8.78] 1	0.001	3.82[1.92–7.31] 1	0.001
GCS	Mild Moderate Sever	24 12 90	100 98 54	0.14[0.08–0.25] 0.07[0.04–0.14] 1	0.001 0.001	0.19[0.09–0.43] 0.08[0.04–0.18] 1	0.001 0.001
Shock	Yes No	82 44	82 170	3.86[2.40–6.06] 1	0.001	6.8[3.24–14.14] 1	0.001
Multi organ failure	Yes No	27 99	13 239	5.01[2.48–10.1] 1	0.001	2.69[1.03–6.98] 1	0.042

## Discussion

The study has tried to identify determinants of maternal mortality among ICU-admitted patients of WSUCSH by including some important variables. Five factors were recognized as predictors of mortality: length of stay in the ICU, level of consciousness on admission, which is expressed in the Glasgow Coma Scale (GCS), being on invasive mechanical ventilation, having shock, and multi-organ failure as complications.

Odds of maternal mortality was nearly 3 time [AOR=2.61, 95% CI: 1.41, 4.82] higher for obstetric patients who stayed in ICU for <5 days as compared to those stayed in ICU for more than 5 days. This result aligns with an ICU studies conducted in Northern, Western and Northwestern Ethiopia [32–34]. The populations' similar traits may explain this likeness. But it contradicts a study conducted in Turkey [35]. The impact of premature death can be linked to delays in the search for urgent care, resulting in life-threatening complications. WHO also had declared the main determinants of maternal mortality in the third world, the first being failures in the health system, which is interpreted as delays in seeking care and delays in receiving care after reaching the health care facility [23, 32]. The dissimilarity with Turkeys study might be a consequence of difference in level of ICU setup, time of care initiation or continuum of care, level of quality service, divergent management or follow up approaches and difference in study area or setting which is third world in our case. All these may settle on determinant factors. 

Odds of maternal mortality was about eighty percent [AOR=0.19, 95% CI: 0.09, 0.43] less for mildly (13-15) decreased level of consciousness (GCS) on admission and Ninety Two Percent [AOR=0.08, 95% CI: 0.04, 0.18] less for moderately (9-12) decreased level of consciousness on admission as compared to severely decreased level of consciousness. It’s similar with the finding of studies conducted in Ethiopia (Addis Ababa and Northern Ethiopia) [17, 34] and Malawi [2]. This can be justified as patients with a severely decreased level of consciousness (coma). During periods of unconsciousness, patients lose their protective reflexes and sensory responses, rendering them vulnerable to aspiration, anoxic brain injuries, airway obstruction, and skin ulcerations [36].

Odds of maternal mortality is nearly four times higher among those on Invasive Mechanical ventilation as compared to with those participants who were not supported by the machine. The finding is compatible with studies conducted in Northwest Ethiopia [32], Nigeria [2], Brazil [1] and Indian [37]. The condition can be vindicated as, compared to developed countries, patients on mechanical ventilation in developing countries have a higher death rate. This may be due to a due to a lack of awareness, using used or donated equipment, or inadequate maintenance [38]. Despite its benefits, patients on invasive mechanical ventilation are at risk of significant yet potential preventable complications like pulmonary embolism, pneumothorax, or ventilation-associated pneumonia even emotional stressed and mental health problems [39].

Odds of maternal mortality was nearly seven time [AOR=6.8, 95% CI: 3.24, 14.14] higher for obstetric patients whom developed shock in ICU in comparison with obstetric patients who were not diagnosed with shock as a complication., which is in line with studies conducted in Brazil [1] and Seira Leon [24] and Northern Ethiopia [34]. It can be justified as, any type of shock can result in tissue hypo perfusion, hypoxia and ischemia, can lead to multi-organ failure; if this is not treated in a timely and appropriate manner, it can lead to death.

Odds of maternal mortality was nearly three times [AOR=2.69, 95% CI: 1.03, 6.98] higher for mothers whom developed Multi-Organ Failure as compared to those obstetric patients who weren’t developed MOF. The finding is supported by Northern Ethiopian [40] and Nigerian [2] and South African [41] studies. It may be narrated as patients, admitted to ICU present with a variety of life-threatening complications that result in a sequential dysfunction of organs and systems, which is considered the main cause of death in ICUs worldwide [34]. Patients, especially those with vital organ failure like the heart, lung, or brain, may experience difficulty with gas exchange, deprived tissue perfusion, and loss of consciousness, ultimately resulting in cell death. 

The study tries to analyze the impact of multiple factors on obstetric mortality, and depending on the findings, the study has implications for policymakers and stakeholders to focus on ICU quality care by providing necessary logistics, trained manpower, and evidence-based strategies. There will also be implications for senior researchers to conduct studies by including some behavioral factors, social or psychological support, issues with transportation and referring systems, as well as issues that are difficult to observe, like professional care delivery systems and equipment functionality. It may be a survival analysis or observational study.

## Strengths and limitations of the study

Several possible risk factors for an outcome of interest are measured using the case-control study design, and the chart document offers a range of expert viewpoints and views.

By its very nature, a retrospective or chart review study has concerns with measuring some important variables that may not be included in that chart, such as behavioral factors, social or psychological support, transportation and referral issues and trouble observing professional care.

## Conclusions

To sum up, this study emphasized finding potential causes of maternal mortality in the intensive care unit. The evidence presented in this paper found that the following factors could predict maternal mortality at Wolaita Sodo Comprehensive Specialized Hospital. These are decreased length of stay in the ICU, reduced level of consciousness (GCS) up on admission, use of invasive mechanical ventilation, having developed shock complications, and developing multi-organ failures.

## Recommendations

For Wolaita Sodo University Comprehensive Specialized Hospital (WSUCSH):

- Enhanced the development of shock prevention protocols and standardized ICU admission criteria.
- The ICU is encouragedto develop early warning scores to prevent and forecast complications
- The Hospital is enhanced to use alternative oxygen delivery methods over mechanical ventilator, recommended to facilitate training on the suitable use of mechanical ventilation and better to check functionality of mechanical ventilators.

For WSUCSH Health professionals:

- Health professionals are suggested to use a neurological observation chart to follow patients admitted with decreased level of consciousness
- Health providers are recommended to conduct specific organ function tests to early diagnose and treat multi-organ failures

For Researchers:

- Lastly, researchers are recommended to verify findings like decreases length of stay in ICU and increased odds of mortality using survival or time-to-event analysis.
- Researchers are recommended to carryout prospective cohort study by including additional key variables like behavioural, psychological and lifestyle factors.

## Data Availability

Raw data were generated at Wolaita Sodo University Comprehensive specialized Hospital. Derived data supporting the findings of this study are available from the Tigist Nega Alemu, School of Public Health, Wolaita Sodo University, Sodo City, Ethiopia, email: tiginega88@gmail.com on request.

## Abbreviations

ANC: Antenatal care
GCS: Glasgow coma scale
HEELP: Hemolysis elevated liver enzymes and low platelets
ICD: International statistical classification of diseases and related health problems
ICU: Intensive care unit
IMV: Invasive mechanical ventilation
MOF: Multi-organ failure
PPH: Post partum hemorrhage
WSUCSH: Wolaita sodo university comprehensive specialized

## References

[1] Saintrain SV, Oliveira JG, Saintrain MV, Bruno ZV, Borges JL, Daher EF, Silva GB Jr. Factors associated with maternal death in an intensive care unit. Rev Bras Ter Intensiva. 2016;28(4):397–404. 28099637 10.5935/0103-507X.20160073 PMC5225914

[2] Adeniran ASo B, O.Fawole AA. Predictors of maternal mortality among critically ill obstetric patients. Malawi Med J. 2015;27(1):16. 26137193 10.4314/mmj.v27i1.5 PMC4478400

[3] Bauserman M, Thorsten VR, Nolen TL et al. Maternal mortality in six low and lower-middle income countries from 2010 to 2018: risk factors and trends. Reprod Health 2020;17(173). 10.1186/s12978-020-00990-z PMC7745363 33334343

[4] Lima HM, Carvalho FH, Feitosa FE, Nunes GC. Factors associated with maternal mortality among patients meeting criteria of severe maternal morbidity and near miss. Int J Gynaecol Obstet. 2017;136(3):337–43. 28099693 10.1002/ijgo.12077

[5] Demsash AWWA. Women’s health service access and associated factors in Ethiopia: application of geographical information system and multilevel analysis. BMJ Health Care Inform. 2023. 10.1136/bmjhci-2022-100720. 37116949 10.1136/bmjhci-2022-100720 PMC10151888

[6] Yihune Teshale M, Bante A, Gedefaw Belete A et al. Barriers and facilitators to maternal healthcare in East Africa: a systematic review and qualitative synthesis of perspectives from women, their families, healthcare providers, and key stakeholders. BMC Pregnancy Childbirth. 2025);25(111). 10.1186/s12884-025-07225-8 PMC11792318 39901111

[7] Bhavanam N, Munisamaiah M, Jayalingegowda. Profile of obstetric patients in intensive care unit -a retrospective study. Int J Reprod Contracept Obstet Gynecol. 2023;12(3):619–22.

[8] Ismail S, Sohaib M. Obstetric patients requiring critical care: retrospective study in a tertiary care institute of Pakistan. J Obstetric Anaesth Crit Care. 2019;9(2):56.

[9] Yi HYJSKS, Kim Y, Choi SJ, Oh SY, Roh CR, Kim JH. Indications and characteristics of obstetric patients admitted to the intensive care unit: a 22-year review in a tertiary care center. Obstet Gynecol Sci. 2018;61(2):209–19. 29564311 10.5468/ogs.2018.61.2.209 PMC5854900

[10] Ozumba BCAL, Obi VO, Umeh UA, Enebe JT, Obioha KC. Pattern and outcome of obstetric admissions into the intensive care unit of a southeast Nigerian hospital. Indian J Crit Care Med. 2018;22(1):16–9. 29422727 10.4103/ijccm.IJCCM_297_17 PMC5793016

[11] Surekha T, Neha G, Poonam S, Dinesh B, Apurva R, Himanshu B, Jaya K. Role of obstetric high dependency and intensive care unit in improving pregnancy outcome and reducing maternal Mortality-A study in rural central India. Int J Crit Care Emerg Med 2018;4(2).

[12] Walls A, Plaat F, Delgado AM. Maternal death: lessons for anaesthesia and critical care. BJA Educ. 2022;22(4):146–53. 35531079 10.1016/j.bjae.2021.11.009 PMC9073293

[13] Okafor UVAU. Admission pattern and outcome in critical care obstetric patients. Int J Obstet Anesth. 2004;13:164–6. 15321395 10.1016/j.ijoa.2004.04.002

[14] Mideksa T, Mekonnen T, Mengiste B. Outcomes and Associated Factors of Mothers Admitted to Intensive Care Unit During Pregnancyand Postpartum at Saint Paul’s Hospital Millennium Medical College, Addis Ababa, Ethiopia. Res Square 2022.

[15] Tesfay N, Tariku R, Zenebe A, Habtetsion M, Woldeyohannes F. Place of death and associated factors among reviewed maternal deaths in Ethiopia: a generalised structural equation modelling. BMJ Open. 2023;13(1):e060933. 36697051 10.1136/bmjopen-2022-060933 PMC9884926

[16] Shiferaw MA, Bekele D, Surur F, Dereje B, Tolu LB. Maternal death review at a tertiary hospital in Ethiopia. Ethiop J Health Sci. 2021;31(1):35–42. 34158750 10.4314/ejhs.v31i1.5 PMC8188111

[17] Tasew A, Melese E, Jemal S, Getachew L. Obstetrics mortality and associated factors in intensive care unit of addis Ababa public hospital in, 2020/21: A hospital based case control study. Annals Med Surg 2022;81. 10.1016/j.amsu.2022.104458 PMC9486713 36147061

[18] Ugochukwu VO. EREaAA: risk factors for maternal deaths in unplanned obstetric admissions to the intensive care unit-lessons for Sub-Saharan Africa. Afr J Reprod Health. 2011;15(4):53. 22571105

[19] Sultan PAN, Rhodes A. Provision of critical care services for the obstetric population. Best Pract Res Clin Obstet Gynaecol. 2013;27:803–9. 23972289 10.1016/j.bpobgyn.2013.07.005

[20] Rudakemwa A, Cassidy AL, Twagirumugabe T. High mortality rate of obstetric critically ill women in Rwanda and its predictability. BMC Pregnancy Childbirth. 2021. 10.1186/s12884-021-03882-7. 34034687 10.1186/s12884-021-03882-7 PMC8144868

[21] Abie AGMM, Eseyneh Dagnew T, et al. Obstetric admission and maternal mortality in the intensive care unit in Africa: a systematic review and meta-analysis. PLoS One. 2025. 10.1371/journal.pone.0320254. 40238732 10.1371/journal.pone.0320254 PMC12002433

[22] Chan GJDJ, Getnet M, et al. Gaps in maternal, newborn, and child health research: a scoping review of 72 years in Ethiopia. J Glob Health Rep. 2021;5: e2021033.

[23] Organization WH, UNFPA. Trends in maternal mortality 2000 to 2020: estimates by WHO, UNICEF. Geneva: World Bank Group and UNDESA/Population Division; 2023.

[24] Marotta CP, Gennaro LD, Cavallin F, Bah F. Epidemiology, outcomes, and risk factors for mortality in critically ill women admitted to an obstetric high-dependency unit in Sierra Leone. Am J Trop Med Hyg. 2020;103(5):2142–8. 32840199 10.4269/ajtmh.20-0623 PMC7646769

[25] Chen Y, Shi J, Zhu Y, Kong X, Lu Y, Chu Y, Mishu MM. Women with maternal near-miss in the intensive care unit in Yangzhou, China: a 5-year retrospective study. BMC Pregnancy Childbirth. 2021;21(1):784. 34798869 10.1186/s12884-021-04237-y PMC8602992

[26] WHO. Strategies towards ending preventable maternal mortality. Geneva, Swetherland: WHO; 2015.

[27] Gebretsadik A, Tarekegne Z, Teshome M. Retrospective review of maternal deaths in Hawassa comprehensive specialised hospital, in southern Ethiopia. J Obstet Gynaecol. 2020;40(5):659–65. 31512545 10.1080/01443615.2019.1648398

[28] Hussein J, Hirose A, Owolabi O, Imamura M, Kanguru L, Okonofua F. Maternal death and obstetric care audits in Nigeria: a systematic review of barriers and enabling factors in the provision of emergency care. Reprod Health. 2016;13:47. 27102983 10.1186/s12978-016-0158-4 PMC4840864

[29] Mimani Minuta W, Lera T, Haile D, Badacho AS, Bekele B, Gezume Ganta A, Nigussie Bolado G, Bashe B. Lived experience of mothers having preterm newborns in a neonatal intensive care unit at Wolaita Sodo university comprehensive specialized hospital Southern ethiopia: a phenomenological study. Res Rep Neonatol. 2023;13:1–14.

[30] World Health Organization. The WHO application of ICD-10 to deaths during pregnancy, childbirth and the puerperium: ICD-MM. Geneva: World Health Organization; 2012. p. 66. Report No.: WHO/RHR/12.05.

[31] bede FMGYM. Incidence and predictors of mortality among patients admitted to adult intensive care unit at public hospitals in Western Ethiopia: a retrospective cohort study. Front Med. 2024;11(1370729). 10.3389/fmed.2024.1370729 PMC11614645 39635586

[32] Demass TB, Guadie AG, Mengistu TB et al. The magnitude of mortality and its predictors among adult patients admitted to the Intensive care unit in Amhara Regional State, Northwest Ethiopia. Sci Rep. 2023;13(12010). 10.1038/s41598-023-39190-7 PMC10368686 37491467

[33] Melaku EEUB, Dessie F, Seid A, Abebe Z, Tefera AS. Determinants of mortality of patients admitted to the intensive care unit at Debre Berhan comprehensive specialized hospital: a retrospective cohort study. Patient Relat Outcome Meas. 2024;15:61–70. 38410832 10.2147/PROM.S450502 PMC10895994

[34] Demirkiran O, Dikmen Y, Utku T, Urkmez S. Critically ill obstetric patients in the intensive care unit. Int J Obstet Anesth. 2003;12(4):266–70. 15321455 10.1016/S0959-289X(02)00197-8

[35] Plum FPJ. The diagnosis of stupor and coma. Contemp Neurol Ser. 1972;10:1–286. 4664014

[36] Kumar R, Gupta A, Suri T, Suri J, Mittal P, Suri JC. Determinants of maternal mortality in a critical care unit: a prospective analysis. Lung India. 2022;39(1):44–50. 34975052 10.4103/lungindia.lungindia_157_21 PMC8926236

[37] Debebe F, Goffi A, Haile T, Alferid F, Estifanos H, Adhikari NKJ. Predictors of ICU mortality among mechanically ventilated patients: an inception cohort study from a tertiary care center in addis ababa, Ethiopia. Crit Care Res Pract. 2022;2022:7797328. 36533249 10.1155/2022/7797328 PMC9754825

[38] Alemayehu M, Azazh A, Hussien H, Baru A. Characteristics and outcomes of mechanically ventilated patients at adult ICU of selected public hospitals in Addis Ababa, Ethiopia. Open Access Emerg Med. 2022;14:395–404. 35942403 10.2147/OAEM.S369752 PMC9356700

[39] Yohannes T, yibrah BerheZelelow, Adhana MT. Obstetric ICU admissions and their outcomes in AyderComprehensive Specialized Hospital: Institution based retrospective study. East Afr J HealthSci. [cited 2025 Aug. 1]. 2020;2(1):250–62. 10.71624/k9vhm908.

[40] Buga EC MC, FCOG (SA), Nethathe GD. MB chb, DA (SA) etal: obstetric critical care services in South Africa. S Afr J Obstet Gynaecol. 2015;21(1):4–5.

[41] Melberg A, Mirkuzie AH, SisayTA, Sisay MM, Moland KM. 'Maternal deaths should simply be0': politicization of maternal death reporting and reviewprocesses in Ethiopia. Health Policy Plan. 2019;34(7):492-8.10.1093/heapol/czz075. 10.1093/heapol/czz075 PMC6788214 31365076

